# Axes of social inequities in COVID-19 clinical trials: A systematic review

**DOI:** 10.3389/fpubh.2023.1069357

**Published:** 2023-02-14

**Authors:** Anna Ponjoan, Constanza Jacques-Aviñó, Laura Medina-Perucha, Victor Romero, Ruth Martí-Lluch, Lia Alves-Cabratosa, Rafel Ramos, Anna Berenguera, María del Mar Garcia-Gil

**Affiliations:** ^1^Grup en Salut Vascular de Girona (ISV-Girona), Institut Universitari d'Investigació en Atenció Primària (IDIAPJGol), Girona, Spain; ^2^Institut d'Investigació Biomèdica de Girona Dr. Josep Trueta (IDIBGI), Girona, Spain; ^3^Universitat Autònoma de Barcelona, Bellaterra, Spain; ^4^Institut Universitari d'Investigació en Atenció Primària (IDIAPJGol), Barcelona, Spain; ^5^Servicio Canario de la Salud, Santa Cruz de Tenerife, Spain; ^6^Department of Medical Sciences, School of Medicine, Universitat de Girona, Girona, Spain; ^7^Department of Nursing, Universitat de Girona, Girona, Spain

**Keywords:** SARS-CoV-2, intersectionality, social determinants of health, disparities, ethnic and racial minorities, sexual and gender minorities, LGBT

## Abstract

**Objective:**

The representativeness of participants is crucial to ensure external validity of clinical trials. We focused on the randomized clinical trials which assessed COVID-19 vaccines to assess the reporting of age, sex, gender identity, race, ethnicity, obesity, sexual orientation, and socioeconomic status in the results (description of the participants' characteristics, loss of follow-up, stratification of efficacy and safety results).

**Methods:**

We searched the following databases for randomized clinical trials published before 1st February 2022: PubMed, Scopus, Web of Science, and Excerpta Medica. We included peer-reviewed articles written in English or Spanish. Four researchers used the Rayyan platform to filter citations, first reading the title and abstract, and then accessing the full text. Articles were excluded if both reviewers agreed, or if a third reviewer decided to discard them.

**Results:**

Sixty three articles were included, which assessed 20 different vaccines, mainly in phase 2 or 3. When describing the participants' characteristics, all the studies reported sex or gender, 73.0% race, ethnicity, 68.9% age groups, and 22.2% obesity. Only one article described the age of participants lost to follow-up. Efficacy results were stratified by age in 61.9%, sex or gender in 26.9%, race and/or, ethnicity in 9.5%, and obesity in 4.8% of the articles. Safety results were stratified by age in 41.0%, and by sex or gender in 7.9% of the analysis. Reporting of gender identity, sexual orientation or socioeconomic status of participants was rare. Parity was reached in 49.2% of the studies, and sex-specific outcomes were mentioned in 22.9% of the analysis, most of the latter were related to females' health.

**Conclusions:**

Axes of social inequity other than age and sex were hardly reported in randomized clinical trials that assessed COVID-19 vaccines. This undermines their representativeness and external validity and sustains health inequities.

## 1. Introduction

Historically, the adequate inclusion of certain populations in clinical trials has been insufficient, compromising the generalizability of results and enhancing health inequities (1). The analysis of the vaccines' efficacy and safety, in particular, is related to the innate and adaptive immunity (2), which differs not only by sex or age (3) but also by other axes of social inequity (ASI) In this regard, stress, depression, and anxiety can hinder the immune response. In the context of the COVID19 pandemic, anxiety was more prevalent among those affected by food insecurity, which, in turn, is related to lower socioeconomic status (4). Similarly, disadvantaged populations (women, Asians, Hispanics, foreign-born, elderly people, and sexual and gender minorities) are more likely to feel stress and depression (5, 6), and consequently, vaccine efficacy may be reduced in these persons. Moreover, elderly people (7), those with obesity (8), and populations living in poverty (9) have shown higher risk of infection, hospitalization, and death; and women were more exposed to the virus, because they commonly assumed, as has usually been the case, caregiving tasks (10).

In view of the potential differences in the efficiency and safety of COVID19 vaccination in these populations, clinical trials should address them but only sex and/or gender and age have been partially examined so far (2, 11). Therefore, we aimed at analyzing if COVID-19 vaccine trials accounted for axes of social inequities in the description of participant characteristics, follow-up, and results about efficacy and safety. In particular, we focused on those social and demographic factors which alters the access to health care and are involved in health inequities: age, sex, gender identity, sexual orientation, socioeconomic status, race, ethnicity, and obesity (10, 12). We included obesity because its social stigma promotes health barriers (13), and makes communication difficult. For example, women with obesity who participate in cancer screening programs experienced insensitive comments about weight, equipment, and gowns that could not accommodate them (14).

## 2. Methods

This systematic review followed the standards set forth by the PRISMA statement (15).

### 2.1. Eligibility criteria

We included randomized clinical trials aimed at analyzing the efficacy and safety of COVID-19 vaccines. We included trials published before February 1, 2022, conducted in humans, and written in English or Spanish, which are the languages the authors master. We excluded trials with no available abstract, without submission to peer-review, or published as reviews, systematic reviews, meta-analyses, editorials, letters, case reports, comments, short communications, or conference abstracts.

### 2.2. Search strategy and study selection

We searched PubMed, Scopus, Web of Science, and Excerpta Medica Database. Supplementary Table S1 shows the search strategy. The Rayyan Intelligent Systematic Review software was used to manage the studies obtained (16). Four researchers were involved in the screening (CJ-A, LM-P, VR, RM-LL) and each author was paired with all other authors, so that the pairs were exchangeable. First, each study was reviewed by reading both title and abstract. Then, the pre-selected papers were full-text screened for inclusion following the same exchangeable pair-review process. Each study was screened by two different author pairs, one pair screened the title and abstract and the other pair revised the full-text. Articles were included when both reviewers agreed, and discrepancies were resolved by a third reviewer.

### 2.3. Data extraction

From the included articles the following variables were extracted: publication details (author, year of publication, title, journal, funding source); study characteristics (vaccine name, sample size, recruitment's country, trial phase), and ASI (age, sex, gender identity, race, ethnicity, obesity, sexual orientation, and socioeconomic status or education). We focused on the description of participants' characteristics, losses to follow-up, and stratification of results about efficacy and safety. We also checked if the study assessed parity and mentioned sex-specific outcomes. The terminology of some axes of social inequities is controversial and confusing. For example the use of the terms race and ethnicity has long been subject for discussion (17); and sex and gender, which have been considered critical variables in COVID-19 vaccines trials, are frequently used interchangeably even though they play different roles in COVID-19 (11). Therefore, we aimed to describe the terminology used to refer to sex, gender, race and/or ethnicity. We used the Excel software to calculate absolute and relative frequencies of the study variables. The inclusion and exclusion criteria and the data extraction methodology were standardized in a pilot analysis by reviewing five abstracts individually. Then, the reviewers shared their decisions and reached consensus on the abstract exclusion and inclusion criteria. Subsequently, each of them extracted data from one article and agreed on the analysis approach.

## 3. Results

**Figure 1:**
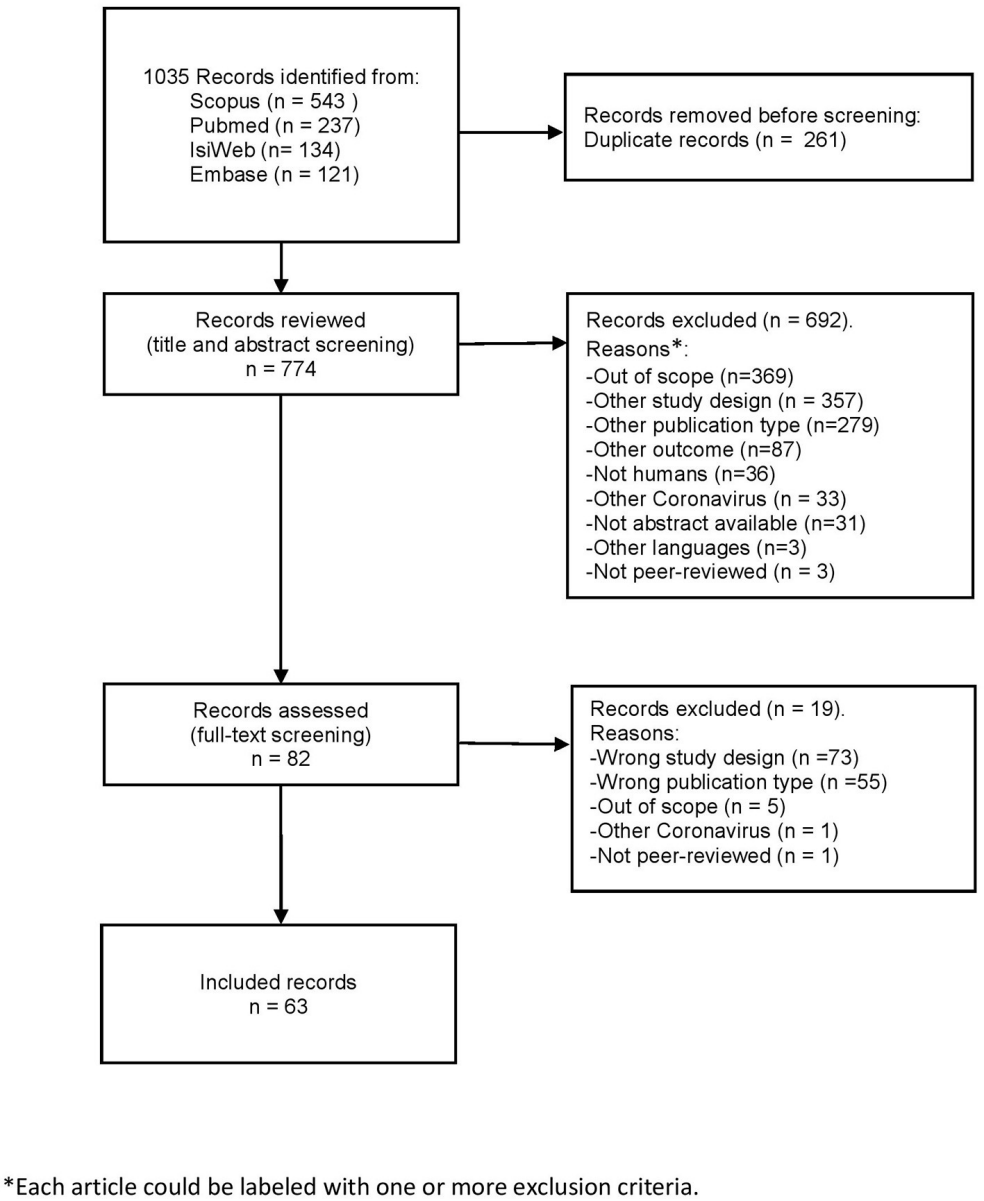
PRISMA guidelines flow chart.

We identified 1,035 citations from the databases, from which 261 were duplicates. We screened 774 citations by reading the title and abstract, and 82 articles by reading the full-text (Figure 1). A total of 711 citations did not fulfill inclusion criteria and were excluded.

Data from 63 trials were included in this review. Characteristics of the studies are detailed in Supplementary Tables S2, S3. Twenty different vaccines were assessed. Mostly, the selected studies evaluated Covishield/Vaxzevria (*n* = 9, 17.3%) and CoronaVac (*n* = 6, 11.5%) vaccines; were published in 2021; were developed in phase 2 or 3; included the general population; and were conducted in Asia, Europe, or North America (Table 1). Globally, sex distribution was reported in all the studies, and age and race and/or ethnicity was reported in about 70% of them (Table 2). Stratification of results on vaccine efficacy was mainly conducted by age groups, and less frequently by sex, race and/or ethnicity, and obesity (Table 3). This stratification was mainly conducted by age groups and less frequently by sex (Table 4). Sexual orientation and socioeconomic status/education attainment were scarcely reported (Tables 3, 4). The percentage of publications assessing ASI by participant characteristics, follow-up, efficacy and safety results, and number of ASI reported are shown in Supplementary Figure S1.

**Table 1:** Description of the characteristics of the 63 included studies.

	**Total**
	***N*** **(%)**
Year of publication 2020	10 (17.5)
2021	47 (74.6)
2022	5 (7.9)
RCT Phase 1	13 (20.6)
2	27 (42.9)
3	19 (30.2)
4	2 (3.2)
Not reported	2 (3.2)
Funding source Public	21 (33.3)
Private	13 (20.6)
Public and Private	28 (44.4)
Not reported	1 (1.6)
Study population General	51 (80.6)
Diagnosed or treated	8 (12.7)
Occupational High-risk	4 (6.4)
Recruitment Content Africa	2 (3.2)
Asia	23 (36.5)
Europe	11 (17.5)
Oceania	2 (3.2)
North America	11 (17.5)
South America	2 (3.2)
International collaboration	12 (19.1)

**Table 2:** Report of participants characteristics by axes of social inequity according to study characteristics.

			**Age**	**Sex/ gender Identity**	**Race/ ethnicity**	**Obesity**	**Sexual orientation**	**Socioeconomics/ education**
		***N***	***N*** **(%)**	***N*** **(%)**	***N*** **(%)**	***N*** **(%)**	***N*** **(%)**	***N*** **(%)**
Total		63	44 (68.9)	63 (100)	46 (73.0)	14 (22.2)	0	1 (1.6)
Phase	1	13	9 (69.2)	13 (100)	11 (84.6)	0	0	0
	2	27	17 (69.2)	27 (100)	18 (66.7)	4 (14.8)	0	0
	3	19	17 (89.5)	19 (100)	15 (78.9)	8 (42.1)	0	0
	4	2	1 (50.0)	2 (100)	2 (100)	2 (100)	0	1 (50.0)
	Not reported	2	0	2 (100)	0	0	0	0
Funding	Both	28	22 (78.6)	28 (100)	23 (82.1)	8 (28.6)	0	0
	Private	13	11 (84.6)	13 (100)	10 (76.9)	3 (23.1)	0	0
	Public	21	11 (52.4)	21 (100)	12 (57.1)	3 (14.2)	0	1 (3.5)
	Not reported	1	0	1 (100)	1 (100)	0	0	0
Population general		51	38 (74.5)	51 (100)	31 (72.6)	10 (19.6)	0	0
Diagnosed or treated		8	3 (37.5)	8 (100)	5 (62.5)	3 (37.5)	0	0
High-risk occupational		4	3 (75.0)	4 (100)	4 (100)	1 (25.0)	0	1 (25.0)
Place	Africa	2	1 (50.0)	2 (100)	2 (100)	2 (100)	0	0
	Asia	23	18 (78.3)	23 (100)	11 (47.8)	3 (13.0)	0	1 (4.3)
	Oceania	2	1 (50.0)	2 (100)	2 (100)	0	0	0
	Europe	11	4 (36.4)	11 (100)	7 (63.6)	2 (18.2)	0	0
	International	12	12 (100)	12 (100)	11 (91.7)	5 (41.2)	0	0
	North America	11	7 (63.4)	11 (100)	11 (100)	1 (9.1)	0	0
Central/South America		2	1 (50.0)	2 (100)	2 (100)	1 (50.0)	0	0

**Table 3:** Report of efficacy results stratified by axes of social inequity according to study characteristics.

			**Age**	**Sex/ gender identity**	**Race/ ethnicity**	**Obesity**	**Sexual orientation**	**Socioeconomics/ education**
		***N***	***N*** **(%)**	***N*** **(%)**	***N*** **(%)**	***N*** **(%)**	***N*** **(%)**	***N*** **(%)**
Total		63	39 (61.9)	17 (26.9)	6 (9.5)	3 (4.8)	0	0
Phase	1	13	5 (38.5)	1 (7.7)	0	0	0	0
	2	27	16 (59.3)	3 (11.5)	0	1 (3.7)	0	0
	3	19	16 (84.2)	10 (52.6)	6 (31.6)	2 (10.5)	0	0
	4	2	1 (50.0)	2 (100)	0	0	0	0
	Not reported	2	1 (50.0)	1 (50.0)	0	0	0	0
Funding	Both	28	17 (60.7)	6 (21.4)	2 (7.1)	1 (3.6)	0	0
	Private	13	10 (76.9)	2 (16.7)	2 (15.4)	1 (7.7)	0	0
	Public	21	12 (57.1)	9 (42.9)	2 (9.5)	1 (4.8)	0	0
	Not reported	1		0	0	3 (5.9)	0	0
Study population general		51	34 (66.7)	12 (24.0)	4 (7.8)	0	0	0
Diagnosed or treated		8	3 (37.5)	4 (50.0)	2 (25.0)	0	0	0
High-risk occupational		4	2 (50.0)	1 (33.3)	0		0	0
Place	Africa	2	0	0	0	0	0	0
	Asia	23	16 (69.6)	7 (31.8)	0	1 (4.4)	0	0
	Oceania	2	1 (50.0)	0	0	0	0	0
	Europe	11	5 (45.4)	3 (27.3)	1 (9.1)	0	0	0
	International	12	11 (91.7)	4 (33.3)	3 (25)	2 (16.7)	0	0
	North America	11	5 (45.5)	2 (18.2)	2 (18.2)	0	0	0
Central/South America		2	1 (50.0)	1 (50.0)	0	0	0	0

**Table 4:** Report of safety results stratified by axes of social inequity according to study characteristics.

			**Age**	**Sex/ gender Identity**	**Race/ ethnicity**	**Obesity**	**Sexual orientation**	**Socioeconomics/ education**
		***N***	***N*** **(%)**	***N*** **(%)**	***N*** **(%)**	***N*** **(%)**	***N*** **(%)**	***N*** **(%)**
Total		63	25 (41.0)	5 (7.9)	0	0	0	0
Phase	1	13	4 (33.3)	0	0	0	0	0
	2	27	13 (48.2)	2 (7.4)	0	0	0	0
	3	19	8 (47.1)	3 (17.7)	0	0	0	0
	4	2	0	0	0	0	0	0
	Not reported	2	0	0	0	0	0	0
Funding	Both	28	11 (44.0)	3 (12.0)	0	0	0	0
	Private	13	6 (46.2)	0	0	0	0	0
	Public	21	8 (38.1)	2 (9.5)	0	0	0	0
	Not reported	1	0	0	0	0	0	0
Study population General		51	22 (44.0)	3 (6.0)	0	0	0	0
Diagnosed or treated		8	3 (37.5)	2 (25.0)	0	0	0	0
High-risk occupational		4	0	0	0	0	0	0
Place	Africa	2	0	0	0	0	0	0
	Asia	23	12 (52.2)	2 (8.7)	0	0	0	0
	Oceania	2	1 (50.0)	0	0	0	0	0
	Europe	11	2 (20.0)	1 (10.0)	0	0	0	0
	International	12	4 (36.4)	1 (9.1)	0	0	0	0
	North America	11	5 (50.0)	1 (10.0)	0	0	0	0
Central/South America		2	1 (50.0)	0	0	0	0	0

### 3.1. Age

One study analyzed only the pediatric population, 60 included adults with a variety of minimum ages in the inclusion criteria (16, 18, 20, 50, 60 years). Two studies included both pediatric and adult populations (over 12 years of age as an inclusion criterion).

Overall, age was more commonly considered in the description of participants and in the stratification of results (efficacy and safety) in phase 2 or 3 studies, in analysis funded by private sources, focused on the general population, or conducted in several countries or in Asia (Tables 2, 3).

### 3.2. Sex and gender identity

Sex was reported in all the included studies when describing the participants' characteristics, but using mixed terms: 61 studies reported male/female percentages, 59 of which referred to sex and two to gender; two studies used the term sex to describe the percentages of men and women. Three studies described sex with more than two categories: Stephenson et al. (18) used the terms females, males, and undifferentiated (non-binary sex); Sadoff et al. (19) reported females, males, and non-binary; and Sadoff et al. (20) described females, males, non-binary, unknown. Only one trial described both–self-reported-sex (termed male/female) and gender (male, female, transgender women, and transgender men) (21). No trials used the term gender identity.

Regarding representativeness, a total of 31 trials reached parity—defined as women percentages ranging from 45 to 55%. Among the 32 studies without sex-parity, 16 studies included a higher percentage of women and 16 of men. Efficacy results were more frequently stratified by sex than safety results (Tables 3, 4). Efficacy and safety results were more frequently stratified in phase 3–4 studies, studies that received public funding, studied specific populations, and were conducted in Europe, Asia, North America, or through international collaborations (Tables 3, 4). Sixty-one studies assessed safety; of these, 12 analysis (19.6%) considered sex-specific outcomes, 12 mentioned outcomes related to female's health, and 3 to male's health (Supplementary Table S4).

### 3.3. Race and ethnicity

Race and/or ethnicity was considered in 46 studies (73%) in the description of participants' characteristics, and no particular pattern was observed regarding the study phase. This concept was less frequently reported when the study focused on the general population, received public funding, or was conducted in Asia or Europe (Table 2). The groups most frequently described were: White (*n* = 35), Asian (*n* = 30), Black or African American (*n* = 28). Only 17 studies specified that race and/or ethnicity was self-reported by participants. Both ethnicity and race were reported separately in 8 trials, and pooled in 12 trials; the remaining studies reported one single domain: 15 described ethnicity, 8 race, 1 ancestry (22), 1 nationality (23), and provided the percentage of white participants without using any specific term (24). Results were seldom stratified by race or ethnicity, especially those regarding safety. Stratification of results on efficacy was more common in phase-3 studies, in analysis of diagnosed or treated populations, or conducted internationally or in North America (Tables 3, 4).

### 3.4. Obesity

Forty-three studies reported obesity when describing the participants' characteristics, of which 29 described the body mass index as a continuous variable, and 14 described obesity as a categorical variable. Obesity categories were more frequently reported in studies performed in phase 3 or 4, funded by both private and public sources, focused on specific populations, or conducted in Africa or (South, Central or South) America (Table 2). Only three studies stratified results on efficacy by obesity groups and no stratification was found for results about (Tables 3, 4).

## 4. Discussion

This comprehensive review highlights the deficiency of ASI reporting in randomized clinical trials assessing COVID-19 vaccines. The ASI more frequently described were age, sex and race or ethnicity. Obesity, socioeconomic status, and gender identity were hardly evaluated; none of the studies included sexual orientation.

Regarding the participants' characteristics, the percentages of studies reporting age and sex were similar to those in previous reports (2, 11, 25, 26). Our percentages of race and/or ethnicity reporting were higher than the wide range, 8.5–59%, observed in previous trials (27, 28). This could be partially explained by the elevated number of international collaborations included in our analysis, and of studies with sample sizes >200 participants (76%), which was associated with improved reporting of race and/or ethnicity (27). Obesity was reported in 20% of the studies, as in a previous review of COVID-19 clinical trials (29). Socioeconomic status/education attainment level was reported in 1.6% of the trials, which is a much lower percentage than the figures observed in a sample of 100 randomized clinical trials: 23% reported education attainment and 2% income levels (30).

Sex distribution was balanced in half of the studies, and 25% of them underrepresented females, in line with previous analysis (2, 11, 25). The lack of parity observed in many trials requires further consideration right from the study design and recruitment. Furthermore, we also observed a mixed use of the terms sex and gender, in agreement with previous research (25), and no consideration of the gender identity. The vaccines efficacy and safety could vary according not only to sex-specific biological differences in the immune system, genetics, or hormones (31), but also to gender specific differences. Gender is a social construct that includes identity, cultural norms, roles, and behaviors assigned to each sex, which might alter the efficacy and safety of COVID-19 vaccines. For example, the immune response might depend on the diet and access to health care; a previous study showed that in many countries, women tended to have lower access to healthy nutrition and health care services than men due to higher levels of poverty (32).

Self-reporting is recommended when capturing race and ethnicity data (17, 33). Our results showed that race and/or ethnicity was self-reported in about 30%of the studies, a higher percentage than the 9% observed in general medical literature (17). Regarding terminology, most of the trials used the term “ethnicity,” which is being increasingly used in biological sciences (34). The term “race” was still commonly used although it is being considered obsolete, and researchers call for the elimination of the use of race in biological sciences (35). Terminology in the field of race and ethnicity remains for continuous investigation and critical reflection (34). In our sample, all trials conducted in the USA reported race and/or ethnicity data. This might be explained by the public policies and guidelines from the National Health Institute to enhance the participation of racial and ethnic groups and other minorities in clinical trials (36). However, reporting does not necessarily imply better representativeness, as suggested by a recent study that found underrepresentation of Black and Asian groups in trials about COVID-19 prevention (37).

The representativeness of a trial not only depends on who is included, but also on who completed the follow-up (38). Our study showed an alarming lack of ASI reporting in relation to participants lost to follow-up, concurring with previous analysis (39, 40). The description of these participants is crucial because the attrition might be biased according to certain ASI; e.g., older persons might be more prone to fall ill and to discontinue the trial (41), or persons with low income might depend on public transport to attend the trial visits (30). If ASI modified inclusion or attrition of participants, both representativeness and external validity might be compromised (42, 43). The stratification of results by ASI is necessary for equitable research (44). In vaccine trials, stratification is important to assess potential differences in efficacy and safety across different populations. Vaccine antibody response is lower in elderly people (26, 45), or in persons with obesity (46–49). In contrast, the immune response is enhanced in cisgender females (50, 51), who could achieve a similar antibody response, with half of the influenza vaccine dose, to that in males (52). Certain hormones and the microbiome seem to play a role in the differences in immunogenicity or safety between cisgender males and females (53); thus, it is plausible that they also alter the immune response or safety in some trans and intersexual persons (54). Adverse effects were more frequent in adults aged < 55 years, but only for certain types of COVID-19 vaccines; whereas older people were more affected by Moderna (55). People with low income are more prone to suffer from multimorbidity and therefore more susceptible to adverse effects related to vaccines (30). Evidence previous to the pandemic showed that adverse effects of vaccines related to autoimmune and allergic reactions might be more frequent and severe in women than in men (31, 53, 55, 56). However, about 80% of the reviewed trials did not specify by sex when reporting health outcomes related to vaccine safety. In this regard, recent studies have suggested that COVID-19 vaccination might affect menstruation, and cause unexpected vaginal bleeding (57–59). The concerning scarcity of stratified results might be partially due to small sample sizes -specially in phase 1 or 2 studies-, although recommendations suggest reporting stratified results also when the sample is small, because they can be used in future meta-analyses (60).

The lack of consideration of ASI contributes to perpetuating health and social inequities in different ways. First, invisibilization prevents the generation of evidence for specific groups, and therefore, COVID-19 policies or clinical practice might exclude these people or implement strategies without enough scientific evidence. For example, age was poorly reported in clinical trials that examined the effects of vaccination, but elderly were one of the first groups to be vaccinated in many countries (61). Second, invisibilization might hinder adequate training of healthcare professionals maintaining inequitable health care practices. Third, invisibilization might bolster vaccination indecision in underrepresented individuals, such as older persons living alone (62), women (63), and sexual and gender minorities (64, 65). Hesitancy regarding COVID-19 vaccination in certain groups might reduce vaccine effectiveness in the general population. The lack of consideration of ASI in COVID-19 vaccine trials could be partially explained by the urgent necessity to develop effective vaccines amid the pandemic. The development of these vaccines had to be conducted in an extremely short period, which may have complicated the adequate consideration of ASI.

We acknowledge some limitations in our analysis. First, we could not discard a potential selection bias since we only included articles written in English or Spanish. However, it is unlikely the results were biased as most of the published literature is written in English or Spanish. Second, we assessed representativeness of sex but not of other ASI, such as race or ethnicity, because specific national population distributions were not available. Second, other ASI such as religion, functional diversity, or language were not evaluated. Further research is needed to assess ASI applying an intersectional approach, to identify barriers to trials' access and completeness of follow-up, and to assess if it improves equity in including participants. This would result in more accurate evidence on effectiveness of vaccination, which in turn would help achieve higher rates of participation in clinical trials and of vaccination acceptance amongst the hesitant population.

## 5. Conclusions

Randomized clinical trials assessing COVID-19 vaccines hardly considered ASI. Only sex and age were commonly reported when describing the participants' characteristics or stratifying efficacy results; the specification of other ASI was rare. The lack of representativeness of certain groups in clinical trials enhances their invisibility and perpetuates health inequities.

## Data availability statement

The original contributions presented in the study are included in the article/Supplementary material, further inquiries can be directed to the corresponding author.

## Author contributions

MG-G, RR, AB, and AP conceptualized the research and found funding. MG-G and AP monitored all the steps of the research. CJ-A, LM-P, VR, and RM-L conducted the searches, filtering process and data extraction. LA-C wrote the first draft of the manuscript. All authors contributed to interpreting the results and writing the article.
