# Supplementary material for: Axes of social inequities in COVID-19 clinical trials: A systematic review
Source: Front Public Health. 2023 Feb 14;11:1069357. doi: 10.3389/fpubh.2023.1069357 (PMC9987589; doi:10.3389/fpubh.2023.1069357)

Table S1. Search terms used in each database.

Database	Search
Pubmed	(((safe* OR "side effect"[Title/Abstract] OR "adverse event"[Title/Abstract] OR "adverse effect"[Title/Abstract] OR "adverse reaction"[Title/Abstract] OR "adverse outcome"[Title/Abstract] OR "undesirable effect"[Title/Abstract] OR effectiveness)) AND ((COVID-19 OR SARS-CoV-2 OR Coronavirus) AND (vaccin*[Title/Abstract]))) AND (("clinical trial"[Title/Abstract] OR "randomized controlled trial"[Title/Abstract])) Filters: Humans, English, Spanish, from 2020 - 2021 Sort by: Publication Date
Isi Web of Knowledge	((TS=(COVID-19 OR SARS-CoV-2 OR Coronavirus)) AND ((TI=(vaccine*)) OR (AB=(vaccine*))) AND (TI=("clinical trial" OR "randomized controlled trial")) OR (AB=("clinical trial" OR "randomized controlled trial"))) AND ((TI=(safe* OR "side effect" OR "adverse event" OR "adverse effect" OR "adverse reaction" OR "adverse outcome" OR "undesirable effect" OR effectiveness)) OR (AB=(safe* OR "side effect" OR "adverse event" OR "adverse effect" OR "adverse reaction" OR "adverse outcome" OR "undesirable effect" OR effectiveness)))
Scopus	(ALL (covid-19 OR sars-cov-2 OR coronavirus) AND TITLE-ABS-KEY (vaccine*) AND TITLE-ABS-KEY ("clinical trial" OR "randomized controlled trial") AND TITLE-ABS-KEY ((safe* OR "side effect" OR "adverse event" OR "adverse effect" OR "adverse reaction" OR "adverse outcome" OR "undesirable effect" OR effectiveness))) AND (LIMIT-TO (PUBYEAR , 2021) OR LIMIT-TO (PUBYEAR , 2020)) AND (LIMIT-TO (DOCTYPE , "ar")) AND (LIMIT-TO (LANGUAGE , "English") OR LIMIT-TO (LANGUAGE , "Spanish")) AND (LIMIT-TO (SRCTYPE , "j"))
Excerpta Medica Database (EMBASE)	('covid 19' OR 'sars cov 2' OR coronavirus) AND vaccine*:ab,ti AND ('clinical trial':ab,ti OR 'randomized controlled trial':ab,ti) AND (safe*:ab,ti OR 'side effect':ab,ti OR 'adverse event':ab,ti OR 'adverse effect':ab,ti OR 'adverse reaction':ab,ti OR 'adverse outcome':ab,ti OR 'undesirable effect':ab,ti OR effectiveness:ab,ti)

Table S2. References of the 63 articles included in the review.

Baden LR, Sahly HME, Essink B, et al. Efficacy and Safety of the mRNA-1273 SARS-CoV-2 Vaccine. *N Engl J Med*. 2021;384(5):403-416. doi:10.1056/nejmoa2035389

Barrett JR, Belij-Rammerstorfer S, Dold C, et al. Phase 1/2 trial of SARS-CoV-2 vaccine ChAdOx1 nCoV-19 with a booster dose induces multifunctional antibody responses. *Nat Med*. 2021;27(2):279-288. doi:10.1038/s41591-020-01179-4

Bonelli M, Mrak D, Tobudic S, et al. Additional heterologous versus homologous booster vaccination in immunosuppressed patients without SARS-CoV-2 antibody seroconversion after primary mRNA vaccination: a randomised controlled trial. *Ann Rheum Dis*. 2022;81(5):687-694. doi:10.1136/annrheumdis-2021-221558

Borobia AM, Carcas AJ, Pérez-Olmeda M, et al. Immunogenicity and reactogenicity of BNT162b2 booster in ChAdOx1-S-primed participants (CombiVacS): a multicentre, open-label, randomised, controlled, phase 2 trial. *The Lancet*. 2021;398(10295):121-130. doi:10.1016/S0140-6736(21)01420-3

Bueno SM, Abarca K, González PA, et al. Safety and Immunogenicity of an Inactivated Severe Acute Respiratory Syndrome Coronavirus 2 Vaccine in a Subgroup of Healthy Adults in Chile. *Clin Infect Dis*. Published online September 2021. doi:10.1093/cid/ciab823

Chappell KJ, Mordant FL, Li Z, et al. Safety and immunogenicity of an MF59-adjuvanted spike glycoprotein-clamp vaccine for SARS-CoV-2: a randomised, double-blind, placebo-controlled, phase 1 trial. *Lancet Infect Dis*. 2021;21(10):1383-1394. doi:10.1016/S1473-3099(21)00200-0

Chu L, McPhee R, Huang W, et al. A preliminary report of a randomized controlled phase 2 trial of the safety and immunogenicity of mRNA-1273 SARS-CoV-2 vaccine. *Vaccine*. 2021;39(20):2791-2799. doi:10.1016/j.vaccine.2021.02.007

Ella R, Reddy S, Blackwelder W, et al. Efficacy, safety, and lot-to-lot immunogenicity of an inactivated SARS-CoV-2 vaccine (BBV152): interim results of a randomised, double-blind, controlled, phase 3 trial. *The Lancet*. 2021;398(10317):2173-2184. doi:10.1016/S0140-6736(21)02000-6

Ella R, Vadrevu KM, Jogdand H, et al. Safety and immunogenicity of an inactivated SARS-CoV-2 vaccine, BBV152: a double-blind, randomised, phase 1 trial. *Lancet Infect Dis*. 2021;21(5):637-646. doi:10.1016/S1473-3099(20)30942-7

Ella R, Reddy S, Jogdand H, et al. Safety and immunogenicity of an inactivated SARS-CoV-2 vaccine, BBV152: interim results from a double-blind, randomised, multicentre, phase 2 trial, and 3-month follow-up of a double-blind, randomised phase 1 trial. *Lancet Infect Dis*. 2021;21(7):950-961. doi:10.1016/S1473-3099(21)00070-0

Emary KRW, Golubchik T, Aley PK, et al. Efficacy of ChAdOx1 nCoV-19 (AZD1222) vaccine against SARS-CoV-2 variant of concern 202012/01 (B.1.1.7): an exploratory analysis of a randomised controlled trial. *The Lancet*. 2021;397(10282):1351-1362. doi:10.1016/S0140-6736(21)00628-0

Falsey AR, Sobieszczyk ME, Hirsch I, et al. Phase 3 Safety and Efficacy of AZD1222 (ChAdOx1 nCoV-19) Covid-19 Vaccine. *N Engl J Med*. 2021;385(25):2348-2360. doi:10.1056/nejmoa2105290

Feng Y, Chen J, Yao T, et al. Safety and immunogenicity of inactivated SARS-CoV-2 vaccine in high-risk occupational population: a randomized, parallel, controlled clinical trial. *Infect Dis Poverty*. 2021;10(1). doi:10.1186/s40249-021-00924-2

Folegatti PM, Ewer KJ, Aley PK, et al. Safety and immunogenicity of the ChAdOx1 nCoV-19 vaccine against SARS-CoV-2: a preliminary report of a phase 1/2, single-blind, randomised controlled trial. *The Lancet*. 2020;396(10249):467-478. doi:10.1016/S0140-6736(20)31604-4

Formica N, Mallory R, Albert G, et al. Different dose regimens of a SARS-CoV-2 recombinant spike protein vaccine (NVXCoV2373) in younger and older adults: A phase 2 randomized placebo-controlled trial. *PLoS Med*. 2021;18(10). doi:10.1371/journal.pmed.1003769

Frenck RW, Klein NP, Kitchin N, et al. Safety, Immunogenicity, and Efficacy of the BNT162b2 Covid-19 Vaccine in Adolescents. *N Engl J Med*. 2021;385(3):239-250. doi:10.1056/nejmoa2107456

Goepfert PA, Fu B, Chabanon AL, et al. Safety and immunogenicity of SARS-CoV-2 recombinant protein vaccine formulations in healthy adults: interim results of a randomised, placebo-controlled, phase 1–2, dose-ranging study. *Lancet Infect Dis*. 2021;21(9):1257-1270. doi:10.1016/S1473-3099(21)00147-X

Guo W, Duan K, Zhang Y, et al. Safety and immunogenicity of an inactivated SARS-CoV-2 vaccine in healthy adults aged 18 years or older: A randomized, double-blind, placebo-controlled, phase 1/2 trial. *EClinicalMedicine*. 2021;38. doi:10.1016/j.eclinm.2021.101010

Halperin SA, Ye L, MacKinnon-Cameron D, et al. Final efficacy analysis, interim safety analysis, and immunogenicity of a single dose of recombinant novel coronavirus vaccine (adenovirus type 5 vector) in adults 18 years and older: an international, multicentre, randomised, double-blinded, placebo-controlled phase 3 trial. *The Lancet*. 2022;399(10321):237-248. doi:10.1016/S0140-6736(21)02753-7

Han B, Song Y, Li C, et al. Safety, tolerability, and immunogenicity of an inactivated SARS-CoV-2 vaccine (CoronaVac) in healthy children and adolescents: a double-blind, randomised, controlled, phase 1/2 clinical trial. *Lancet Infect Dis*. 2021;21(12):1645-1653. doi:10.1016/S1473-3099(21)00319-4

Heath PT, Galiza EP, Baxter DN, et al. Safety and Efficacy of NVX-CoV2373 Covid-19 Vaccine. *N Engl J Med*. 2021;385(13):1172-1183. doi:10.1056/nejmoa2107659

Hsieh SM, Liu MC, Chen YH, et al. Safety and immunogenicity of CpG 1018 and aluminium hydroxide-adjuvanted SARS-CoV-2 S-2P protein vaccine MVC-COV1901: interim results of a large-scale, double-blind, randomised, placebo-controlled phase 2 trial in Taiwan. *Lancet Respir Med*. 2021;9(12):1396-1406. doi:10.1016/S2213-2600(21)00402-1

Jackson LA, Anderson EJ, Rouphael NG, et al. An mRNA Vaccine against SARS-CoV-2 — Preliminary Report. *N Engl J Med*. 2020;383(20):1920-1931. doi:10.1056/nejmoa2022483

Kaabi NA, Zhang Y, Xia S, et al. Effect of 2 Inactivated SARS-CoV-2 Vaccines on Symptomatic COVID-19 Infection in Adults: A Randomized Clinical Trial. *JAMA - J Am Med Assoc*. 2021;326(1):35-45. doi:10.1001/jama.2021.8565

Keech C, Albert G, Cho I, et al. Phase 1–2 Trial of a SARS-CoV-2 Recombinant Spike Protein Nanoparticle Vaccine. *N Engl J Med*. 2020;383(24):2320-2332. doi:10.1056/nejmoa2026920

Kremsner PG, Mann P, Kroidl A, et al. Safety and immunogenicity of an mRNA-lipid nanoparticle vaccine candidate against SARS-CoV-2 : A phase 1 randomized clinical trial. *Wien Klin Wochenschr*. 2021;133(17-18):931-941. doi:10.1007/s00508-021-01922-y

Kremsner PG, Guerrero RAA, Arana-Arri E, et al. Efficacy and safety of the CVnCoV SARS-CoV-2 mRNA vaccine candidate in ten countries in Europe and Latin America (HERALD): a randomised, observer-blinded, placebo-controlled, phase 2b/3 trial. *Lancet Infect Dis*. 2022;22(3):329-340. doi:10.1016/S1473-3099(21)00677-0

Li J, Hui A, Zhang X, et al. Safety and immunogenicity of the SARS-CoV-2 BNT162b1 mRNA vaccine in younger and older Chinese adults: a randomized, placebo-controlled, double-blind phase 1 study. *Nat Med*. 2021;27(6):1062-1070. doi:10.1038/s41591-021-01330-9

Liu X, Shaw RH, Stuart ASV, et al. Safety and immunogenicity of heterologous versus homologous prime-boost schedules with an adenoviral vectored and mRNA COVID-19 vaccine (Com-COV): a single-blind, randomised, non-inferiority trial. *The Lancet*. 2021;398(10303):856-869. doi:10.1016/S0140-6736(21)01694-9

Logunov DY, Dolzhikova IV, Shcheblyakov DV, et al. Safety and efficacy of an rAd26 and rAd5 vector-based heterologous prime-boost COVID-19 vaccine: an interim analysis of a randomised controlled phase 3 trial in Russia. *The Lancet*. 2021;397(10275):671-681. doi:10.1016/S0140-6736(21)00234-8

Madhi SA, Baillie V, Cutland CL, et al. Efficacy of the ChAdOx1 nCoV-19 Covid-19 Vaccine against the B.1.351 Variant. *N Engl J Med*. 2021;384(20):1885-1898. doi:10.1056/nejmoa2102214

Madhi SA, Koen AL, Izu A, et al. Safety and immunogenicity of the ChAdOx1 nCoV-19 (AZD1222) vaccine against SARS-CoV-2 in people living with and without HIV in South Africa: an interim analysis of a randomised, double-blind, placebo-controlled, phase 1B/2A trial. *Lancet HIV*. 2021;8(9):e568-e580. doi:10.1016/S2352-3018(21)00157-0

Medeiros-Ribeiro AC, Aikawa NE, Saad CGS, et al. Immunogenicity and safety of the CoronaVac inactivated vaccine in patients with autoimmune rheumatic diseases: a phase 4 trial. *Nat Med*. 2021;27(10):1744-1751. doi:10.1038/s41591-021-01469-5

Meng FY, Gao F, Jia SY, et al. Safety and immunogenicity of a recombinant COVID-19 vaccine (Sf9 cells) in healthy population aged 18 years or older: two single-center,

randomised, double-blind, placebo-controlled, phase 1 and phase 2 trials. *Signal Transduct Target Ther*. 2021;6(1). doi:10.1038/s41392-021-00692-3

Mulligan MJ, Lyke KE, Kitchin N, et al. Phase I/II study of COVID-19 RNA vaccine BNT162b1 in adults. *Nature*. 2020;586(7830):589-593. doi:10.1038/s41586-020-2639-4

Pan HX, Liu JK, Huang BY, et al. Immunogenicity and safety of a severe acute respiratory syndrome coronavirus 2 inactivated vaccine in healthy adults: Randomized, double-blind, and placebo-controlled phase 1 and phase 2 clinical trials. *Chin Med J (Engl)*. 2021;134(11):1289-1298. doi:10.1097/CM9.0000000000001573

Polack FP, Thomas SJ, Kitchin N, et al. Safety and Efficacy of the BNT162b2 mRNA Covid-19 Vaccine. *N Engl J Med*. 2020;383(27):2603-2615. doi:10.1056/nejmoa2034577

Pu J, Yu Q, Yin Z, et al. The safety and immunogenicity of an inactivated SARS-CoV-2 vaccine in Chinese adults aged 18–59 years: A phase I randomized, double-blinded, controlled trial. *Vaccine*. 2021;39(20):2746-2754. doi:10.1016/j.vaccine.2021.04.006

Ramasamy MN, Minassian AM, Ewer KJ, et al. Safety and immunogenicity of ChAdOx1 nCoV-19 vaccine administered in a prime-boost regimen in young and old adults (COV002): a single-blind, randomised, controlled, phase 2/3 trial. *The Lancet*. 2020;396(10267):1979-1993. doi:10.1016/S0140-6736(20)32466-1

Reindl-Schwaighofer R, Heinzel A, Mayrdorfer M, et al. Comparison of SARS-CoV-2 Antibody Response 4 Weeks After Homologous vs Heterologous Third Vaccine Dose in Kidney Transplant Recipients: A Randomized Clinical Trial. *JAMA Intern Med*. 2022;182(2):165-171. doi:10.1001/jamainternmed.2021.7372

Richmond P, Hatchuel L, Dong M, et al. Safety and immunogenicity of S-Trimer (SCB-2019), a protein subunit vaccine candidate for COVID-19 in healthy adults: a phase 1, randomised, double-blind, placebo-controlled trial. *The Lancet*. 2021;397(10275):682-694. doi:10.1016/S0140-6736(21)00241-5

Ryzhikov AB, Ryzhikov E, Bogryantseva MP, et al. A single blind, placebo-controlled randomized study of the safety, reactogenicity and immunogenicity of the “EpiVacCorona” vaccine for the prevention of COVID-19, in volunteers aged 18–60 years (Phase I–II). *Russ J Infect Immun*. 2021;11(1):283-296. doi:10.15789/2220-7619-ASB-1699

Sablerolles RSG, Rietdijk WJR, Goorhuis A, et al. Immunogenicity and Reactogenicity of Vaccine Boosters after Ad26.COV2.S Priming. *N Engl J Med*. 2022;386(10):951-963. doi:10.1056/nejmoa2116747

Sadoff J, Gars ML, Shukarev G, et al. Interim Results of a Phase 1–2a Trial of Ad26.COV2.S Covid-19 Vaccine. *N Engl J Med*. 2021;384(19):1824-1835. doi:10.1056/nejmoa2034201

Sadoff J, Gray G, Vandebosch A, et al. Safety and Efficacy of Single-Dose Ad26.COV2.S Vaccine against Covid-19. *N Engl J Med*. 2021b;384(23):2187-2201. doi:10.1056/nejmoa2101544

Sahly HME, Baden LR, Essink B, et al. Efficacy of the mRNA-1273 SARS-CoV-2 Vaccine at Completion of Blinded Phase. *N Engl J Med*. 2021;385(19):1774-1785. doi:10.1056/nejmoa2113017

Shinde V, Bhikha S, Hoosain Z, et al. Efficacy of NVX-CoV2373 Covid-19 Vaccine against the B.1.351 Variant. *N Engl J Med*. 2021;384(20):1899-1909. doi:10.1056/nejmoa2103055

Shu YJ, He JF, Pei RJ, et al. Immunogenicity and safety of a recombinant fusion protein vaccine (V-01) against coronavirus disease 2019 in healthy adults: a randomized, double-blind, placebo-controlled, phase II trial. *Chin Med J (Engl)*. 2021;134(16):1967-1976. doi:10.1097/CM9.0000000000001702

Sridhar S, Joaquin A, Bonaparte MI, et al. Safety and immunogenicity of an AS03-adjuvanted SARS-CoV-2 recombinant protein vaccine (CoV2 preS dTM) in healthy adults: interim findings from a phase 2, randomised, dose-finding, multicentre study. *Lancet Infect Dis*. 2022;22(5):636-648. doi:10.1016/S1473-3099(21)00764-7

Stephenson KE, Gars ML, Sadoff J, et al. Immunogenicity of the Ad26.COV2.S Vaccine for COVID-19. *JAMA - J Am Med Assoc*. 2021;325(15):1535-1544. doi:10.1001/jama.2021.3645

Tanriover MD, Doğanay HL, Akova M, et al. Efficacy and safety of an inactivated whole-virion SARS-CoV-2 vaccine (CoronaVac): interim results of a double-blind, randomised, placebo-controlled, phase 3 trial in Turkey. *The Lancet*. 2021;398(10296):213-222. doi:10.1016/S0140-6736(21)01429-X

Voysey M, Clemens SAC, Madhi SA, et al. Safety and efficacy of the ChAdOx1 nCoV-19 vaccine (AZD1222) against SARS-CoV-2: an interim analysis of four randomised controlled trials in Brazil, South Africa, and the UK. *The Lancet*. 2021;397(10269):99-111. doi:10.1016/S0140-6736(20)32661-1

Voysey M, Clemens SAC, Madhi SA, et al. Single-dose administration and the influence of the timing of the booster dose on immunogenicity and efficacy of ChAdOx1 nCoV-19 (AZD1222) vaccine: a pooled analysis of four randomised trials. *The Lancet*. 2021;397(10277):881-891. doi:10.1016/S0140-6736(21)00432-3

Walsh EE, Frenck RW, Falsey AR, et al. Safety and Immunogenicity of Two RNA-Based Covid-19 Vaccine Candidates. *N Engl J Med*. 2020;383(25):2439-2450. doi:10.1056/nejmoa2027906

Ward BJ, Gobeil P, Séguin A, et al. Phase 1 randomized trial of a plant-derived virus-like particle vaccine for COVID-19. *Nat Med*. 2021;27(6):1071-1078. doi:10.1038/s41591-021-01370-1

Wu S, Huang J, Zhang Z, et al. Safety, tolerability, and immunogenicity of an aerosolised adenovirus type-5 vector-based COVID-19 vaccine (Ad5-nCoV) in adults: preliminary report of an open-label and randomised phase 1 clinical trial. *Lancet Infect Dis*. 2021;21(12):1654-1664. doi:10.1016/S1473-3099(21)00396-0

Wu Z, Hu Y, Xu M, et al. Safety, tolerability, and immunogenicity of an inactivated SARS-CoV-2 vaccine (CoronaVac) in healthy adults aged 60 years and older: a randomised, double-blind, placebo-controlled, phase 1/2 clinical trial. *Lancet Infect Dis*. 2021;21(6):803-812. doi:10.1016/S1473-3099(20)30987-7

Xia S, Duan K, Zhang Y, et al. Effect of an Inactivated Vaccine Against SARS-CoV-2 on Safety and Immunogenicity Outcomes: Interim Analysis of 2 Randomized Clinical Trials. *JAMA - J Am Med Assoc*. 2020;324(10):951-960. doi:10.1001/jama.2020.15543

Xia S, Zhang Y, Wang Y, et al. Safety and immunogenicity of an inactivated SARS-CoV-2 vaccine, BBIBP-CorV: a randomised, double-blind, placebo-controlled, phase 1/2 trial. *Lancet Infect Dis*. 2021;21(1):39-51. doi:10.1016/S1473-3099(20)30831-8

Zakarya K, Kutumbetov L, Orynbayev M, et al. Safety and immunogenicity of a QazCovid-in® inactivated whole-virion vaccine against COVID-19 in healthy adults: A single-centre, randomised, single-blind, placebo-controlled phase 1 and an open-label phase 2 clinical trials with a 6 months follow-up in Kazakhstan. *eClinicalMedicine*. 2021;39. doi:10.1016/j.eclinm.2021.101078

Zhang Y, Zeng G, Pan H, et al. Safety, tolerability, and immunogenicity of an inactivated SARS-CoV-2 vaccine in healthy adults aged 18–59 years: a randomised, double-blind, placebo-controlled, phase 1/2 clinical trial. *Lancet Infect Dis*. 2021;21(2):181-192. doi:10.1016/S1473-3099(20)30843-4

Zhang J, Hu Z, He J, et al. Safety and immunogenicity of a recombinant interferon-armed RBD dimer vaccine (V-01) for COVID-19 in healthy adults: a randomized, double-blind, placebo-controlled, Phase I trial. *Emerg Microbes Infect*. 2021b;10(1):1589-1597. doi:10.1080/22221751.2021.1951126

Zhu FC, Guan XH, Li YH, et al. Immunogenicity and safety of a recombinant adenovirus type-5-vectored COVID-19 vaccine in healthy adults aged 18 years or older: a randomised, double-blind, placebo-controlled, phase 2 trial. *The Lancet*. 2020;396(10249):479-488. doi:10.1016/S0140-6736(20)31605-6

Table S3. Characteristics of the 63 articles included in the review.

First Author	Year	Vaccine	Phase	Funding	Study Population	Country	Sample size
Baden	2020	Spikevax	3	Public	Diagnosed or treated	USA	30,420
Barrett	2021	Covishield/Vaxzevria	1,2	Both	General	UK	52
Chappell	2021b	Not specified	1	Both	General	Australia	120
Chu	2021	Spikevax	2	Both	General	USA	600
El Sahly	2021	Spikevax	3	Public	Diagnosed or treated	USA	30,415
Ella	2021	Covaxin	1	Private	General	India	375
Emary	2021	Covishield/Vaxzevria	2,3	Both	High-risk occupations	UK	8,534
Folegatti	2020	Covishield/Vaxzevria	1,2	Both	General	UK	1,077
Guo	2021	Not specified	1,2	Public	General	China	1,120
Jackson	2020	Spikevax	1	Both	General	USA	45
Kaabi	2021	Not specified	3	Both	General	United Arab Emirates	40,411
Keech	2020	NVXCoV2373	1	Private	General	Australia	134
Kremsner	2021	CVnCoV	1	Private	General	International	245
Li	2021	BNT162b1	1	Private	General	China	144
Logunov	2021	Sputnik V	3	Both	General	Russia	21,977
Meng	2021	Not specified	1,2	Both	General	China	1,128
Pu	2021	Not specified	1	Public	General	China	192
Ramasamy	2020	Covishield/Vaxzevria	2,3	Both	General	UK	560
Sablerolles	2022	Janssen	3	Public	High-risk occupations	Netherlands	434
Sadoff	2021	Janssen	1,2	Private	General	International	805
Shu	2021	V-01	2	Private	General	China	880
Sridhar	2022	CoV-2 preS dTM	2	Both	General	International	721
Stephenson	2021	Janssen	1	Both	General	USA	25
Voysey	2021	Covishield/Vaxzevria	1,2 and 3	Both	General & High-risk occupations	International	23,848

Voysey	2021b	Covishield/Vaxzevria	1,2 and 3	Both	General	International	24,422
Wu	2021b	Convidecia/PakVak	1	Public	General	China	130
Zhang	2021	CoronaVac	1,2	Public	General	China	743
Zhang	2021b	V-01	1	Private	General	China	180
Zhu	2020	Convidecia/PakVak	2	Both	General	China	508
Ella	2021c	Covaxin	3	Private	General	India	25,798
Falsey	2021	Covishield/Vaxzevria	3	Both	General	International	32,379
Feng Y	2021	Sinopharm	4	Public	High-risk occupations	China	809
Halperin	2022	Convidecia/PakVak	3	Both	General	International	21,250
Heath	2021	NVXCoV2373	3	Private	General	UK	14,039
Hsieh	2021	MVC-COV1901	2	Both	General	Taiwan	3,844
Kremsner	2021b	CVnCoV	2,3	Both	General	International	39,680
Madhi	2021	Covishield/Vaxzevria	1,2	Both	General	South Africa	2,026
Madhi	2021b	Covishield/Vaxzevria	1,2	Both	Diagnosed or treated	South Africa	161
Medeiros-Ribeiro	2021	CoronaVac	4	Public	Diagnosed or treated	Brazil	1,092
Polack	2020	Comirnaty	2,3	Private	General	International	43,448
Sadoff	2021b	Janssen	3	Both	General	International	44,325
Shinde	2021	NVXCoV2373	2	Both	Diagnosed or treated	South Africa	4,387
Tanriover	2021	CoronaVac	3	Public	General	Turkey	10,218
Bonelli	2022	Comirnaty + Spikevax + Covishield/Vaxzevria	Not reported	Public	Diagnosed or treated	Austria	60
Borobia	2021	Comirnaty + Covishield/Vaxzevria	2	Public	Diagnosed or treated	Spain	676
Bueno	2021	CoronaVac	3	Both	General	Chile	434
Ella	2021b	Covaxin	1,2	Private	General	India	380
Formica	2021	NVXCoV2373	2	Both	General	International	1,283
Frenck	2021	Comirnaty	3	Private	General	International	6,052
Goepfert	2021	CoV-2 preS dTM	1,2	Both	General	USA	441

Han	2021	CoronaVac	1,2	Public	General	China	552
Liu	2021	Comirnaty + Covishield/Vaxzevria	2	Public	General	UK	463
Mulligan	2020	BNT162b1	1,2	Not reported	General	USA	45
Pan	2021	KCONVAC	1,2	Public	General	China	560
Reindl-Schwaighofer	2022	Comirnaty + Spìkevax + Janssen	Not reported	Public	Diagnosed or treated	Austria	296
Richmond	2021	SCB-2019	1	Both	General	Australia	151
Ryzhikov	2021	EpiVacCorona	1,2	Public	General	Russia	100
Walsh	2020	Comirnaty + BNT162b1	1	Private	General	USA	195
Ward	2021	CoVLP	1	Public	General	Canada	180
Wu	2021	CoronaVac	1,2	Public	General	China	422
Xia	2020	Not specified	1,2	Both	General	China	320
Xia	2020b	Sinopharm	1,2	Public	General	China	640
Zakarya	2021	QazVac	1,2	Public	General	Kazakhstan	244

Table S4. Sex-specific outcomes reported in the 63 studies that were or were not associated to the COVID-19 vaccine.

Article	Female outcome	Male outcome
El Sahly et al. 2021	Abortion spontaneous, Ectopic pregnancy, Reproductive system and breast disorders, Pelvic pain, Dysfunctional uterine bleeding, Ovarian cyst, Uterine haemorrhage, Breast pain, Endometrial hyperplasia, Pelvic prolapse, Endometrial cancer	Benign prostatic hyperplasia
Ella et al. 2022c	Ovarian cancer with metastases, abortion incomplete	None
Folegatti et al. 2020	Dysmenorrhoea	None
Halperin et al. 2022	Ruptured ectopic pregnancy	None
Kaabi et al. 2020	Outcomes in pregnancy, childbirth and the puerperium	None
Logunov et al. 2021	Spontaneous abortion, vaginitis, corpus luteum cyst, disorder of the menstrual cycle	Prolonged erection, prostatitis
Madhi et al. 2021	Menstrual cycle and uterine bleeding disorders; vulvovaginal disorders; pregnancy, puerperium and perinatal conditions; abortions and stillbirth; uterine.<br><br>Uterine, pelvic and broad ligament disorders	None

Ramasamy et al. 2020	Ovarian cyst	Prostate cancer
Tanriover et al. 2021	Breast cancer; Ovarian cyst	None
Ward et al. 2021	Bacterial vaginosis, vaginal infection, dysmenorrhea, vulvovaginal pruritus	None
Voysey et al. 2021	Uterine leiomyoma, dysmenorrhoea, endometriosis, haemorrhagic ovarian cyst, ovarian germ cell teratoma benign, ovulation pain, uterine haemorrhage, vaginal haemorrhage	None
Voysey et al. 2021b	Breast cancer, Endometriosis and different Reproductive system and breast disorders, Abortion incomplete, Abortion spontaneous, Adnexal torsion, Endometriosis, Genital pain, Haemorrhagic ovarian cyst, Uterine haemorrhage, Vaginal haemorrhage, Ovarian adenoma, Menorrhagia, Uterine haemorrhage Grade Vaginal haemorrhage Grade, Vulvovaginal dryness	None

Figure S1. Percentage of publications assessing axes of social inequity (ASI) by participant characteristics, follow-up, efficacy and safety results, and number of ASI reported.

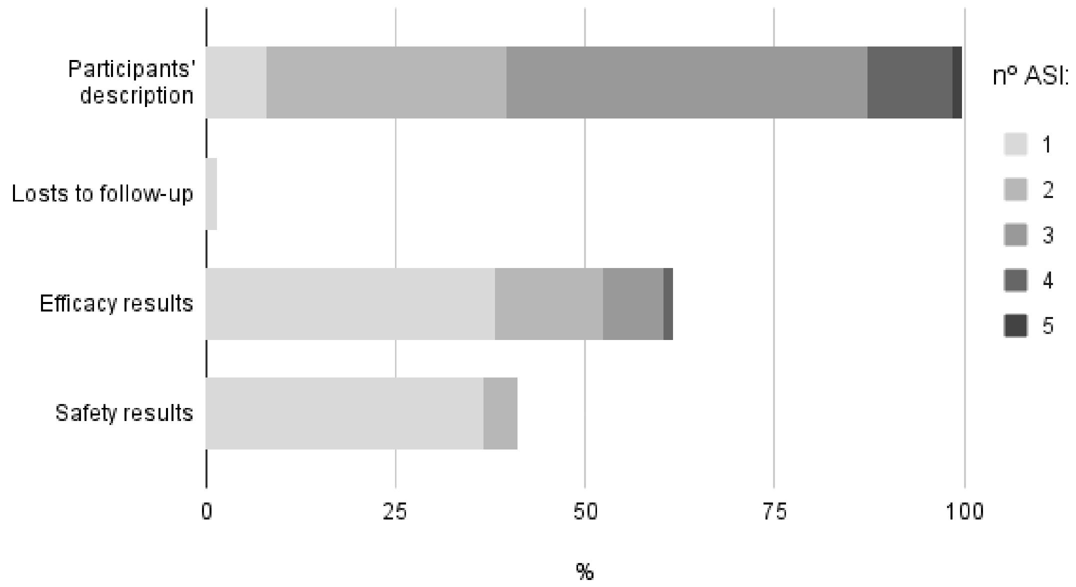

Supplement: Supplementary file 1 [file Data_Sheet_1.pdf]
